# Frondoside A Attenuates Amyloid-β Proteotoxicity in Transgenic *Caenorhabditis elegans* by Suppressing Its Formation

**DOI:** 10.3389/fphar.2020.553579

**Published:** 2020-09-10

**Authors:** Taweesak Tangrodchanapong, Prasert Sobhon, Krai Meemon

**Affiliations:** Department of Anatomy, Faculty of Science, Mahidol University, Bangkok, Thailand

**Keywords:** amyloid-β, oligomers, Alzheimer’s disease, frondoside A, *Caenorhabditis elegans*

## Abstract

Oligomeric assembly of Amyloid-β (Aβ) is the main toxic species that contribute to early cognitive impairment in Alzheimer’s patients. Therefore, drugs that reduce the formation of Aβ oligomers could halt the disease progression. In this study, by using transgenic *Caenorhabditis elegans* model of Alzheimer’s disease, we investigated the effects of frondoside A, a well-known sea cucumber *Cucumaria frondosa* saponin with anti-cancer activity, on Aβ aggregation and proteotoxicity. The results showed that frondoside A at a low concentration of 1 µM significantly delayed the worm paralysis caused by Aβ aggregation as compared with control group. In addition, the number of Aβ plaque deposits in transgenic worm tissues was significantly decreased. Frondoside A was more effective in these activities than ginsenoside-Rg3, a comparable ginseng saponin. Immunoblot analysis revealed that the level of small oligomers as well as various high molecular weights of Aβ species in the transgenic *C. elegans* were significantly reduced upon treatment with frondoside A, whereas the level of Aβ monomers was not altered. This suggested that frondoside A may primarily reduce the level of small oligomeric forms, the most toxic species of Aβ. Frondoside A also protected the worms from oxidative stress and rescued chemotaxis dysfunction in a transgenic strain whose neurons express Aβ. Taken together, these data suggested that low dose of frondoside A could protect against Aβ-induced toxicity by primarily suppressing the formation of Aβ oligomers. Thus, the molecular mechanism of how frondoside A exerts its anti-Aβ aggregation should be studied and elucidated in the future.

## Introduction

Alzheimer’s disease (AD) is a chronic neurodegenerative disorder that affects million people worldwide (Brookmeyer et al., 2007; Masters et al., 2015) and is the most common untreated neurological disease (Citron, 2010). Although Aβ_1–42_ is the most abundant peptide in AD brain, the cause of the disease is due to the formation of numerous Aβ residues (Haass and Selkoe, 2007; Eisenberg and Jucker, 2012). Aβ42 peptide is produced mainly in neurons by proteolytic cleavage of the amyloid precursor protein (LaFerla et al., 2007; Benilova et al., 2012) and self-assembled into fibrillar aggregates, which can be observed in the brains of AD patients (Haass and Selkoe, 2007). Recently, a growing number of evidences revealed that small Aβ oligomers are responsible for the neurotoxicity leading to cognitive impairment in an early stage of AD symptom (Kayed et al., 2003; Nimmrich and Ebert, 2009; Taylor et al., 2010). Therefore, drugs that have ability to suppress amyloid aggregation, particularly during oligomeric assemblies, are required for halting AD progression at the early stage.

**Figure 1:**
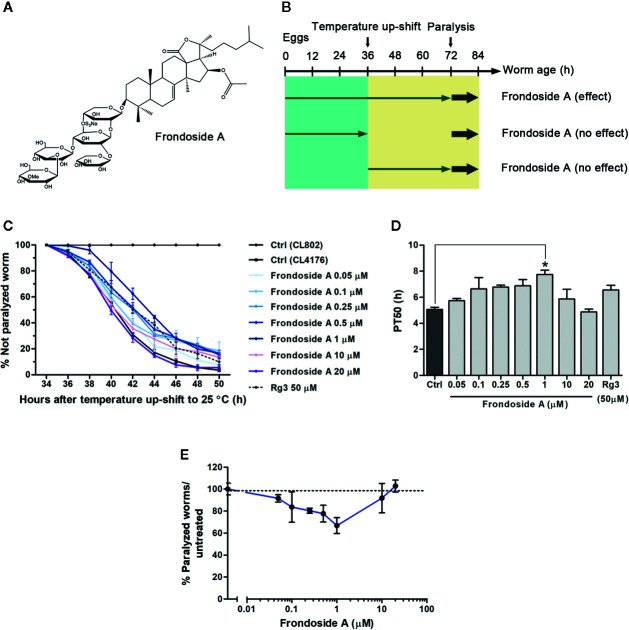
Effect of frondoside A on Aβ-induced paralysis in CL4176 transgenic *C. elegans* strain. **(A)** Structure of frondoside A. **(B)** Diagram illustrating the paralysis assays showing the time at which the temperature was raised in *C. elegans* CL4176 and CL802 (control strain) and duration of worms fed with frondoside A in different treatment plans. **(C)** Time course of Aβ-induced paralysis in transgenic *C. elegans* strains fed with vehicle control (Ctrl) or various concentrations of frondoside A (0.05, 0.1, 0.25, 0.5, 1, 10, and 20 µM) or ginsenoside-Rg3 at 50 µM in all treatment regimen. The paralysis was scored at 2-h intervals. Data are expressed as percentages ± SD of unparalyzed worms from three independent assays with at least 100 worms in each experiment. **(D)** The paralysis index (PT_50_) was quantified as a mean of duration at which 50% worms treated with or without compounds were paralyzed. **(E)** Dose-response effect of frondoside A on Aβ-induced paralysis in CL4176 worms. Error bars indicate SD (*p < 0.05 compared with untreated control CL4176 worms).

Previously, a broad range of ginsenosides, saponins obtained from ginseng herbs, have been widely used for improving cognitive function in AD *via* inhibition of Aβ-induced synaptic loss and neuroinflammation (Kim et al., 2013). Among various ginsenosides, Rg3 compound was found to be the most effective in reducing the pathogenicity of Aβ42 in cultured CHO-2B7 cells as well as in transgenic Tg2576 mice (Chen et al., 2006). Ginsenoside-Rg3 could enhance internalization and phagocytosis of Aβ42 by microglial cells *via* increased expression of macrophage scavenger receptor type A (Joo and Lee, 2005). More recently, relationships between ginsenoside structure and activity against Aβ42 toxicity have been evaluated using transgenic *Caenorhabditis elegans* model of AD (Zhang et al., 2017). In this study, ginsenoside-Rg3, a protopanaxadiol (PPD)-type saponin with sugar moieties attached at C-3 position of the aglycone, was found to significantly reduce Aβ aggregation in the worms. Hence, it is possible that new saponins with similar structure discovered from other species may be more effective for AD treatment. In addition to ginseng herb, sea cucumbers are another major rich source of saponins. Sea cucumber-derived saponins display a wide diversity of biological activities on many types of cells (Kalinin et al., 2005). One of these bioactive compounds is frondoside A (structure shown in **Figure 1A**), a saponin isolated from the sea cucumber *Cucumaria frondosa*. At high concentrations (100 or 800 µg/kg/day), frondoside A could inhibit various biological processes needed by prostate and breast cancer cells for survival without affecting normal cells, body weight, and behaviors in mouse model (Al Marzouqi et al., 2011; Dyshlovoy et al., 2015), thus suggesting its low toxicity. By contrast, subtoxic doses of frondoside A exhibited the divergent effects resulting in cellular activation and potentiation of cellular functions. *In vitro* proteomic analysis demonstrated that frondoside A (0.2 µg/ml or 167 nM) down-regulated heterogeneous nuclear ribonucleoprotein K (HnRNP K) (Aminin et al., 2009), which is involved in switching neurons from proliferation to differentiation (Yano et al., 2005). HnRNP K suppression resulted in translation of p21 mRNA to induce neurite outgrowths from neurons in primary culture (Tanaka et al., 2002), which can be suppressed by the presence of intracellular Aβ oligomers (Umeda et al., 2015). Frondoside A could strongly stimulate lysosomal activity, that has been found to promote Aβ clearance and degradation (Tarasoff-Conway et al., 2015; Chuang et al., 2018), in mouse macrophage *in vivo* when administrated at 0.2 µg per mouse for 10 days and *in vitro* at concentrations of 0.1–0.38 µg/ml (Aminin et al., 2008; Aminin et al., 2010). In AD, oxidative stress is associated with Aβ-induced toxicity (Sultana et al., 2009). Interestingly, frondoside A applied at a concentration of 1 µg/ml also significantly suppressed reactive oxygen species (ROS) in mouse macrophage (Aminin et al., 2008). Therefore, with these properties, we hypothesize that frondoside A at low concentration can attenuate Aβ-induced toxicity by inhibiting its aggregation.

To shorten the time-consuming by using mammalian models and compensate the lack of organismal complexity involved in AD of cell culture, *C. elegans*, a simple organism with a high degree of differentiation (Artal-Sanz et al., 2006), is turned to become a powerful model for *in vivo* AD drug screening (Lublin and Link, 2013). As well, the benefits of multiple AD transgenic strains created in *C. elegans* brought many studies to use them as AD model for testing the efficacy of several nutraceuticals against Aβ42 toxicity (Sangha et al., 2012; Zhang et al., 2016; Zhang et al., 2017). In this study, three transgenic strains are available: The first is CL4176, which expresses human Aβ_1–42_ in muscle cells under a temperature-inducible condition (Link et al., 2003) that results in rapid paralysis. In this strain, the acute induction of the transgene results in the production of Aβ oligomers, which is responsible for the toxicity (Wu et al., 2006; Diomede et al., 2010; Wu et al., 2010). The second *C. elegans* strain, CL2006, constitutively expresses Aβ_3–42_ in muscle cells of the body wall (McColl et al., 2009), leading to an age-related progressive reduction of motility resulted from the accumulation of both fibrils and oligomers of Aβ_3–42_. In addition to the two strains with Aβ expressing in muscle cells, *C. elegans* CL2355 strain has human Aβ transgene engineered into its pan-neuronal cells. The temperature-induced Aβ expression in these neurons causes chemotaxis dysfunction and a defective behavior that can be quantified (Wu et al., 2006). In this study, we have used all these three *C. elegans* strains to investigate the effects of frondoside A treatment on aggregation process and proteotoxicity of Aβ *in vivo*, while treatments with ginsenoside-Rg3 was used as comparable controls.

## Materials and Methods

### Chemicals and Reagents

Frondoside A and ginsenoside-Rg3 were purchased from Sigma-Aldrich (St. Louis, MO, USA). Both compounds were dissolved in dimethyl sulfoxide (DMSO) as a vehicle and stored them at -20°C. For experimental treatments, the stock solutions were directly mixed with *Escherichia coli* strain OP50 as a food source to obtain the final concentration of DMSO at 0.1%. Benzaldehyde, 1,4-bis(3-carboxy-hydroxy-phenylethenyl)-benzene (X-34), and 2′,7′-Dichlorodihydrofluorescein diacetate (H2DCF-DA) were purchased from Sigma-Aldrich (St. Louis, MO, USA).

### *C. elegans* Strains, Maintenance, and Synchronization

The wild-type *C. elegans* strain N2, transgenic strains CL4176 (P*myo-3*::SP::Aβ_1–42_::long 3’ UTR), CL2355 (P*snb-1*::SP::Aβ_1–42_::long 3’ UTR + P*mtl-2*::GFP), CL2006 (P*unc-54*::SP::Aβ_1–42_), CL802 (P*myo-3*; control strain for CL4176), and CL2122 (P*unc-54* + P*mtl-2*::GFP; control strain for CL2355) were obtained from *Caenorhabditis* Genetics Center (University of Minnesota, USA). The expressions of human Aβ_1–42_ in muscle cells of CL4176 and neuronal cells of CL2355 were induced by raising temperature from 16 to 25°C, whereas CL2006 strain constitutively expressed Aβ_3–42_ in muscle cells of the body wall. All *C. elegans* strains were propagated on solid nematode growth medium (NGM) with *E. coli* strain OP50 as a worm food at 16°C. CL2006 and wild-type N2 were maintained at 20°C. To prepare age-synchronized samples, worms were transferred to fresh NGM plates on reaching maturity at 3 days of age and allowed to lay eggs overnight. The synchronized eggs were then isolated and cultured on fresh NGM plates with or without the compounds by using platinum wire at either 16 or 20°C.

### Paralysis Assay

The synchronized eggs of CL4176 and its control CL802 were placed on fresh NGM culture plates containing live *E. coli* strain OP50 mixed with vehicle (0.1% DMSO) or frondoside A at various concentrations (0.05, 0.1, 0.25, 0.5, 1, 10, and 20 µM) at 16°C. Ginsenoside-Rg3 with an effective dose at 50 µM was used as a comparative compound for frondoside A (Zhang et al., 2017). After worms were incubated for 36 h (reaching third-stage larvae), transgene expression of Aβ was induced by temperature up-shifting from 16 to 25°C. An experimental profile of paralysis assay was arranged into three patterns as demonstrated in **Figure 1B**. Pattern 1 (all treatments), synchronized eggs were continuously treated with frondoside A for 36 h at 16°C and after 25°C upshift. Pattern 2 (treatment before temperature upshift), synchronized eggs were treated with frondoside A for 36 h at 16°C and then transferred to the untreated plates. Pattern 3 (treatment after temperature upshift), synchronized eggs were grown on the untreated plates and then treated with frondoside A during 25°C upshift. The worms with no body movement during observation were identified as the paralyzed worms, and they were scored at 2 h intervals until the last worm became paralyzed. In addition to CL4176, CL2006 strain was also selected to perform the paralysis assay. The experiments using CL2006 worms were carried out as previously described by Guo and colleagues (Guo et al., 2016). In brief, synchronized eggs of CL2006 were placed on fresh NGM culture plates seeded with vehicle or various concentrations of frondoside A (0.05–20 µM) at 20°C. After that, the paralyzed worms were scored every day, starting from L4 or young adult stage until all worms were paralyzed. Three independent assays were performed using at least 100 worms in each experiment.

### Western Blotting of Aβ Species

The Aβ species in the transgenic *C. elegans* strains were identified by immunoblotting following the standard Western blotting protocol (Sangha et al., 2012). CL2006 worms treated with or without frondoside A were maintained at 20°C for 96 h. CL4176 worms treated with vehicle or frondoside A were maintained at 16°C for 36 h, then the culture temperature was increased from 16 to 25°C for 36 h. After that, the worms were harvested and washed with ddH_2_O three times. Then, they were boiled in lysis buffer containing 62 mM Tris-HCl (pH 6.8), 2% SDS (w/v), 10% glycerol (v/v), 4% β-mercaptoethanol (v/v), and protease inhibitor cocktail (1X, Sigma-Aldrich, St. Louis, MO, USA) at 105°C for 10 min. After centrifugation at 14,000 g for 5 min, proteins in supernatant were quantified by using Bradford reagent (Bio-Rad, Hercules, CA, USA). Equal amounts of total proteins (30 µg) were boiled prior to electrophoresis for 5 min in denaturation buffer [62 mM Tris-HCl (pH 6.8), 2% SDS (w/v), 10% glycerol (v/v), 4% β-mercaptoethanol (v/v), and 0.0005% bromophenol blue (w/v)]. After heating with denaturation buffer, protein samples were run at 140 V on SDS BIS-Tris gel. The proteins were then transferred from gel to 0.45-µm PVDF membrane for detecting low molecular weights of Aβ species (Sangha et al., 2012) or 0.45-µm nitrocellulose membrane for detecting high molecular weights of Aβ species (Upadhaya et al., 2012) using transferring buffer with 20% methanol. Amyloid protein species were detected with mouse anti-human Aβ1-17 monoclonal antibody clone 6E10 (1:500, MyBioSource, San Diego, CA, USA) and goat anti-mouse lgG-peroxidase conjugated H+L (1:5,000, Sigma-Aldrich, St. Louis, MO, USA). Mouse anti–α-smooth muscle actin antibody, monoclonal clone 1A4, purified from hybridoma cell culture (1:1,000, Sigma-Aldrich, St. Louis, MO, USA) was used to detect actin as an internal control. Bands were detected by enhanced chemiluminescence (ECL, Thermo Fisher Scientific, Waltham, MA, USA) method and imaged with chemiluminescent gel document (Alliance Q9 mini). The mean densities of Aβ immunoreactive bands were analyzed using Image-J software (National Institutes of Health, NIH, Bethesda, MD, USA). Three independent experiments were carried out using approximately 1,000 worms in each group (n ~ 3,000).

### Fluorescent Staining of Aβ Deposits in *C. elegans* Strains

Age-synchronized transgenic strain CL2006 worms were treated with vehicle or frondoside A at 20°C until reaching 120 h of age. The treatment of worms with ginsenoside-Rg3 at 50 µM followed the protocol previously described (Zhang et al., 2017). After treatment, the worms were fixed in 4% paraformaldehyde/phosphate buffered saline (PBS) (pH 7.4) for 24 h at 4°C. After fixation, the worms were stained with 1,4-bis(3-carboxy-hydroxy-phenylethenyl)-benzene (X-34) in 10 mM Tris (pH 8.0) for 4 h at room temperature as described previously (Link et al., 2001). Subsequently, samples were destained with 50% ethanol, mounted on slides, and photographed using a fluorescence microscope (Olympus BX53; Olympus Corp., Tokyo, Japan). Fluorescence images were acquired at the same exposure parameters by using a 40× objective of microscope with a CCD camera. The number of amyloid-reactive deposits was quantified in the worm’s head region anterior to pharyngeal bulb. Data were shown as mean numbers of Aβ deposits/anterior head area of individual worm ± SEM from three independent experiments with 23 worms in each group (n = 69). Wild-type N2 was used as the negative control for transgenic CL2006 strain.

### *Ex Vivo* Measurement of Reactive Oxygen Species (ROS)

Intracellular ROS was measured in transgenic *C. elegans* strains fed with or without the compounds using 2′,7′-Dichlorodihydrofluorescein diacetate (H2DCF-DA) as described previously (Smith and Luo, 2003). After induction with 25°C for 36 h, the worms were collected and washed with PBS + 1% Tween 20 (PBST) three times to remove bacteria. The worms were quickly frozen in liquid nitrogen and then immediately subjected for sonication at equivalent time point to disrupt the outer cuticle. Samples were transferred into wells of black 96-well plates (Thermo Fisher Scientific, Waltham, MA, USA) and incubated with 50 µM H2DCFDA solution (final concentration in PBS) for 30 min at 37°C. Each sampling was carried out in three wells and the assays were independently performed in triplicate using 60 worms in each group. Fluorescent intensity was immediately measured using microplate fluorescence reader Tecan Spark 10M with the excitation at 485 nm and emission at 530 nm.

### Chemotaxis Assay

Chemotaxis assays were performed following the protocol described previously (Margie et al., 2013). Synchronized eggs of transgenic *C. elegans* strain CL2355 and its control strain CL2122 were cultured with or without the compounds at 16°C for 36 h. Then, they were maintained for another 36 h at 25°C to induce neuronal Aβ_1–42_ expression. The worms were collected and washed with M9 buffer three times. Assay was done in 100 mm plates containing 25 mM phosphate buffer (pH 6.0), 1 mM MgSO_4_, 1 mM CaCl_2_, and 1.9% agar. The assay plate was divided into four quadrants with two tests (A&D) and two controls (B&C) (**Figure 6A**). In test areas (A&D), 1 µl of attractant (0.1% benzaldehyde in 100% ethanol) along with 1 µl of 0.25 M sodium azide were added. On the opposite side of the attractant drops, 1 µl of 100% ethanol and sodium azide (1 µl) were added as the control points. Then, the cultured worms were immediately placed at the center of the assay plate. After incubation for 1 h, the number of worms in each quadrant was counted, and the chemotactic index (CI) was calculated. CI value was defined as the number of worms at attractant points - the number of worms at control points/total number of worms. Three independent experiments were done using 60 worms in each group.

### Statistical Analyses

The differences between control and compound-treated groups were statistically compared by one-way ANOVA analysis of variance following the Tukey-Kramer test for multiple comparison results. Percentages of unparalyzed worms were compared using two-way ANOVA. All data analyses were determined by GraphPad Prism software version 5.0. The *p* value < 0.05 was considered as statistically significant.

## Results

### Frondoside A Attenuates Aβ-induced Paralysis in Transgenic *C. elegans*

**Figure 2:**
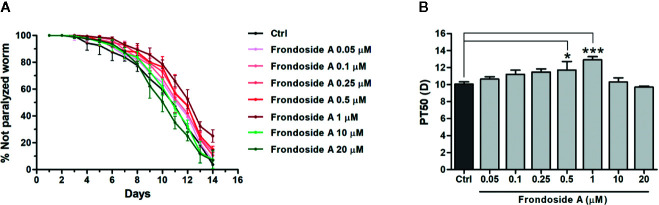
Effect of frondoside A on Aβ-induced paralysis in CL2006 transgenic *C. elegans* strain. **(A)** Time course of paralysis caused by constitutive Aβ_3–42_ expression in CL2006 worms fed with vehicle control (Ctrl) or various concentrations of frondoside A (0.05, 0.1, 0.25, 0.5, 1, 10, and 20 µM). The paralysis was scored every day. Data are expressed as percentages ± SD of unparalyzed worms from three independent assays with at least 100 worms in each experiment. **(B)** The paralysis index (PT50) indicates quantification of a mean time at which 50% worms treated with or without compound were paralyzed. Error bars indicate SD (*p < 0.05 and ***p < 0.001 compared with untreated control CL2006 worms).

To firstly investigate whether frondoside A could attenuate the Aβ-induced toxicity, we administered frondoside A at various concentrations (0.05, 0.1, 0.25, 0.5, 1, 10, and 20 µM) to *C. elegans* strain C4176, which expressed human Aβ in the muscle cells that induced an Aβ-dependent paralysis (Link et al., 2003). Consistent with previous study (Wu et al., 2006), following induction with temperature increase the time-dependent paralysis was observed in CL4176 but not in CL802 worms (control strain with no Aβ expression) (**Figure 1C**). In all treatments from eggs to adult stages, CL4176 worms, frondoside A treatment at concentrations in the range of 0.05–1 µM exhibited an increasing effect in delaying the paralysis phenotype in dose-dependent manner with reaching to the maximum at 1 µM before this effect was declined at higher doses (10 and 20 µM) (**Figure 1C**). This result suggested that frondoside A at low concentrations had the potential to protect against Aβ-induced toxicity. For quantitative analysis, we defined PT_50_ value as mean time interval at which 50% of worms were paralyzed after induction with temperature increase for 36 h. The PT_50_ of worms treated with frondoside A (1 µM) was significantly higher than in vehicle-treated control, whereas no significant difference was observed in worms fed with ginsenoside-Rg3 at 50 µM when compared to the control (**Figure 1D**: Ctrl PT_50_, 5.0 ± 0.5 vs. frondoside A_1 µM_ PT_50_, 7.8 ± 0.6, three experiments, *p* < 0.05 and Ctrl PT_50_ 5.0 ± 0.5 vs. ginsenoside-Rg3_50 µM_ PT_50_ 6.6 ± 0.6, three experiments, *p* > 0.05). The non-significant effect of ginsenoside-Rg3 was also previously reported (Zhang et al., 2017). These results suggested a stronger effect of frondoside A and its concentration at 1 µM was the most effective dose in protecting the worms from Aβ-induced toxicity (**Figure 1E**). However, the feeding of CL4176 with frondoside A before or after the temperature-induced Aβ expression was not able to protect the worms against Aβ-induced paralysis (**Supplementary data**). This implied that short duration treatment with frondoside A might not be sufficient to attenuate Aβ toxicity. In addition to CL4176, we also examined whether frondoside A protected CL2006 worms from the paralysis caused by constitutive Aβ_3–42_ expression. After frondoside A at various concentrations (0.05-20 µM) was administered to CL2006, we observed that its anti-paralytic effect was slightly increased when tested concentration was raised up from 0.05 to 0.5 µM and reached to maximum increase at 1µM compared with untreated control. After that this effect was declined when applied at higher concentrations (10 and 20 µM) (**Figure 2A**). This observation supported that the effect of frondoside A at low concentrations was dose-dependent manner to potentially protect the worms from Aβ-induced toxicity. Consistent with the paralysis result from assays on CL4176, CL2006 worms fed with frondoside A at 1 µM also displayed the highest of the expressing PT_50_ value (mean time interval at which 50% of worms were paralyzed after the worms reached to L4 or young adult stage), which was statistically significant when compared to vehicle-treated worms (**Figure 2B**: Ctrl PT_50_, 10.1 ± 0.3 vs. frondoside A_1 µM_ PT_50_, 12.9 ± 0.4, three experiments, *p* < 0.001). In addition, a significant increase of PT_50_ index was also observed in the worms treated with 0.5 µM frondoside A (**Figure 2B**: Ctrl PT_50_, 10.1 ± 0.3 vs. frondoside A_0.5 µM_ PT_50_, 11.7 ± 1.0, three experiments, *p* < 0.05). These results demonstrated that frondoside A had a wider range of the effective doses (0.5–1 µM) in CL2006, a constitutively Aβ-expressing strain. Thus, all findings suggested that frondoside A at low doses, especially 1 µM, may have the potential to protect against paralysis caused by both inducible and constitutive expression of Aβ in the transgenic *C. elegans* worm strains.

### Frondoside A Affects Aβ Oligomerization in Transgenic *C. elegans*

**Figure 3:**
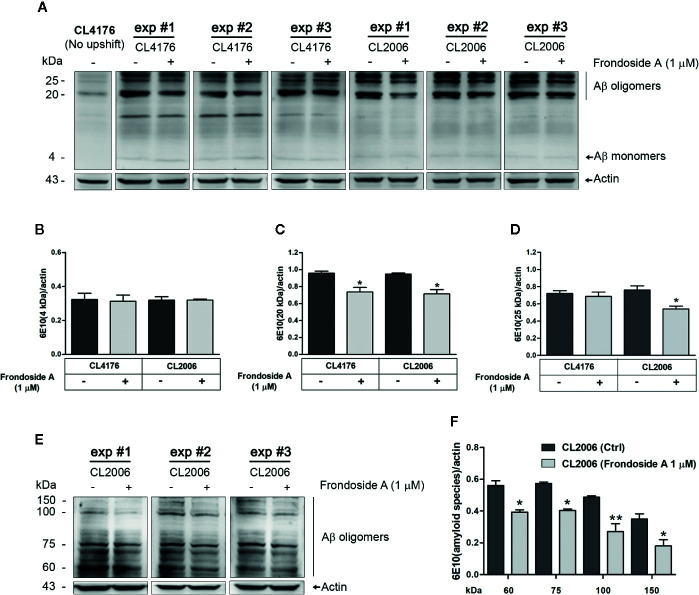
Effect of frondoside A on Aβ species in *C. elegans* strains. **(A)** Representative western blot of low molecular weight Aβ species in CL4176 and CL2006 worms fed with either vehicle or 1 µM frondoside A and detected by anti-Aβ antibody (6E10) or anti-actin. Quantification of Aβ monomers at 4 kDa **(B)**, Aβ oligomers at 20 kDa **(C)**, and 25 kDa **(D)** in both CL4176 and CL2006 fed with frondoside A or vehicle was analyzed by using Image J software. **(E)** Representative western blot of high molecular weights of Aβ species in untreated and treated CL2006 worm tissues as detected by 6E10 antibody. The Aβ oligomer bands at 60, 75, 100, and 150 kDa were quantified **(F)**. The black lines indicate various molecular sizes of Aβ oligomers. The arrows indicate Aβ monomers (4 kDa) or actin (43 kDa). Quantitative data are expressed as mean ± SD of the indicated band density from three independent experiments (exp) with 1,000 worms in each group. *p < 0.05 and **p < 0.01 vs. control group.

To explore the protective mechanism against Aβ-induced paralysis, we analyzed Aβ species from vehicle- or frondoside A–treated transgenic *C. elegans* CL4176 and CL2006 by Western blotting using antibodies against Aβ (6E10). The CL2006 worms were phenotypically characterized by a progressive reduction of motility in correlation with the accumulation of both Aβ_3–42_ fibrils and oligomers. Aβ-immunoreactive (6E10) bands were detected in the tissues from both transgenic worms fed with or without frondoside A, but not in CL4176 proteins from culturing at the permissive temperature (16°C) (**Figures 3A, E**). While the intensity level of Aβ monomer band at 4 kDa in both strains was not altered significantly from the control (**Figure 3B**), the level of Aβ oligomers (the band at 20 kDa) obtained from both CL4176 and CL2006 worms treated with frondoside A at 1 µM was significantly reduced and lower than untreated control for approximately 22.9 and 24.5%, respectively (three independent assays, *p* < 0.05) (**Figure 3C**). Interestingly, the immunoreactive signals of higher molecular weight Aβ oligomer band at 25 kDa did not show a remarkable decrease in CL4176 worms that Aβ expression was induced by the temperature up-shifting; however, this band was significantly dropped in CL2006 that constitutively expressed Aβ upon treatment with 1 µM frondoside A (**Figure 3D**). Thus, we then asked whether frondoside A would also inhibit the formation of high order molecular weight Aβ species in CL2006. Multiple immunoreactive bands of high molecular weights of Aβ species were recognized by 6E10 antibody in both untreated and treated CL2006 worms (**Figure 3E**). As shown in **Figure 3F**, frondoside A-treated worms could significantly reduce mean intensities of various high molecular weights of Aβ oligomer bands at 60, 75, 100, and 150 kDa, which were lesser than untreated control for approximately 29.7% (*p* < 0.05), 29.5% (*p* < 0.05), 44.5% (*p* < 0.01), and 49.1% (*p* < 0.05), respectively. It is likely that frondoside A may initially reduce the level of small Aβ oligomers and then degree of its reducing effect tended to be highly substantial when high molecular weight of Aβ species are formed. These results seem to indicate that the protective effect of frondoside A against Aβ-induced toxicity may be in part of a reduction level of Aβ oligomers as well as the formation of high molecular weight Aβ species in the worms that contributed to delayed onset of paralysis phenotype.

### Frondoside A Inhibits Aβ Deposit in Transgenic *C. elegans*

**Figure 4:**
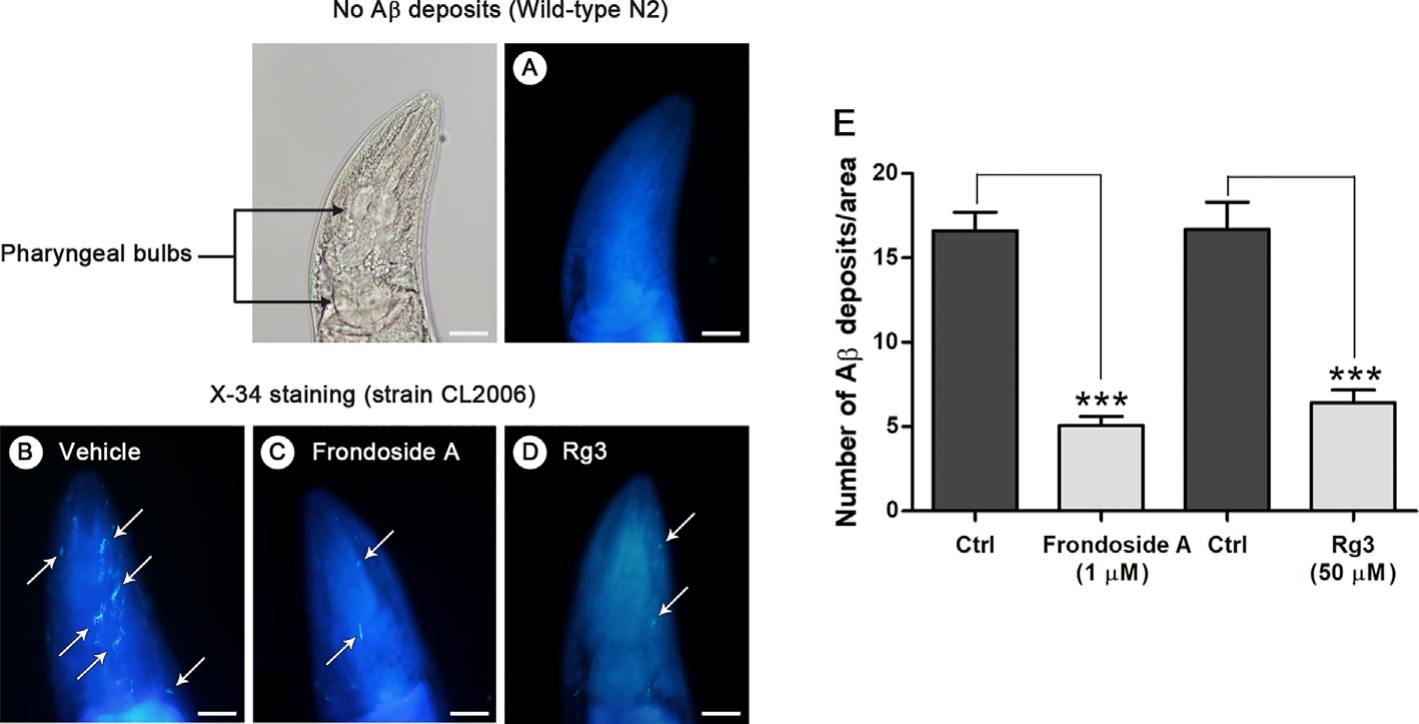
Aβ deposits in transgenic *C. elegans* CL2006 fed with or without frondoside A. Representative images of X-34 staining in the *C. elegans* wild-type N2 **(A)** and transgenic strain CL2006 treated with vehicle **(B)** or frondoside A at 1 µM **(C)** or ginsenoside-Rg3 at 50 µM **(D)**. White arrows indicate Aβ reactive deposits in the worm head, which is separated from body by pharyngeal bulb region (black arrows). Scale bar represents 20 µm. **(E)** Quantitative analysis of Aβ deposits in the head region of transgenic strain CL2006 compared between control (Ctrl) and different treatment groups. The quantity is expressed as mean number ± SEM of Aβ deposits/area of the individual worm’s head region from three different assays with a total of 69 worms (23 worms for each analysis) (***p < 0.001).

*In vitro* study has demonstrated that once oligomers of Aβ are formed, assemblies of amyloid fibril or plaque deposits proceed more readily (Karamanos et al., 2015). Hence, to examine whether frondoside A suppressed Aβ oligomerization, which would prevent formation of amyloid fibril or plaque, *C. elegans* strain CL2006 and wild-type N2, were stained with X-34 fluorescence dye, which recognized Aβ aggregates but not oligomers (Link et al., 2001). Then, the number of Aβ deposits was detected in the head region of the worms. The results showed that amyloid deposits were detected only in CL2006 but not in the wild-type N2 (**Figures 4A–D**). Frondoside A-treated CL2006 worms (at 1 µM) had significantly reduced X-34 positive spots of Aβ deposits when compared to control worms (**Figures 4C, E**). Similarly, CL2006 worms fed with ginsenoside-Rg3 at 50 µM exhibited reduced Aβ deposits (**Figures 4D, E**). These results suggested that the suppressing effect of frondoside A on the formation of Aβ deposits may be due to a reduction level of high molecular weight Aβ species.

### ROS Production Is Alleviated by Frondoside A

**Figure 5:**
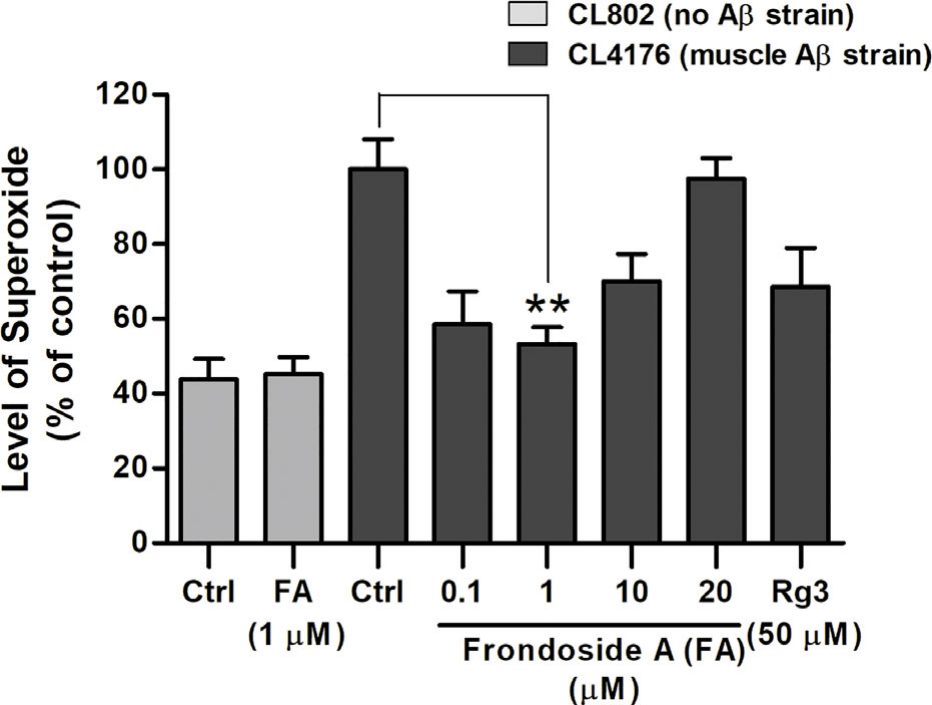
Effect of frondoside A on reactive oxygen species (ROS) in transgenic *C. elegans* fed with vehicle or frondoside A (0.1, 1, 10, and 20 µM) or ginsenoside-Rg3 (50 µM). Synchronized CL4176 and its control strain CL802 maintained at 16°C for 36 h were temperature upshifted to 25°C for 36 h followed by the DCF assay for ROS detection. The representative data are shown as percentage of fluorescence (%DCF) relative to vehicle-treated CL4176 controls (Ctrl), which is set as 100%. Error bars indicate SEM (three independent assays with 60 worms in each group, **p < 0.01 compared with control group).

Significant oxidative stress preceded fibrillar deposition of Aβ, causing phenotypic paralysis of the *C. elegans* strain CL4176 (Drake et al., 2003). Hence, we further investigated whether the reduction of ROS level by frondoside A occurred simultaneously with the reduction of toxic oligomeric species of Aβ. When compared to CL802 strain, which has no Aβ, ROS level was dramatically increased in the untreated CL4176 worms; and the increased ROS level was significantly reduced in CL4176 worms fed with 1 µM frondoside A (100 ± 18.1% for control group and 53.3 ± 7.7% for worms fed with 1 µM frondoside A) (**Figure 5**). Frondoside A at 1 µM exhibited a higher suppressive effect on superoxide production than ginsenoside-Rg3 at 50 µM by about 15% (**Figure 5**). In contrast to CL4176, the ROS level observed in CL802 was not altered when compared between vehicle- and the compound-treated groups as the same experimental condition (**Figure 5**: 43.9 ± 5.4% for control groups, and 45.2 ± 4.6% for CL802 worms fed with 1 µM frondoside A, three experiments, *p* > 0.05). Thus, these results suggested that the ROS reduction by frondoside A may specifically link to Aβ expression and might be in part of its suppressive action on small oligomeric formation of Aβ peptides.

### Frondoside A Suppressed Aβ Expression in Neurons and Prevent Defect in Chemotaxis Behavior

**Figure 6:**
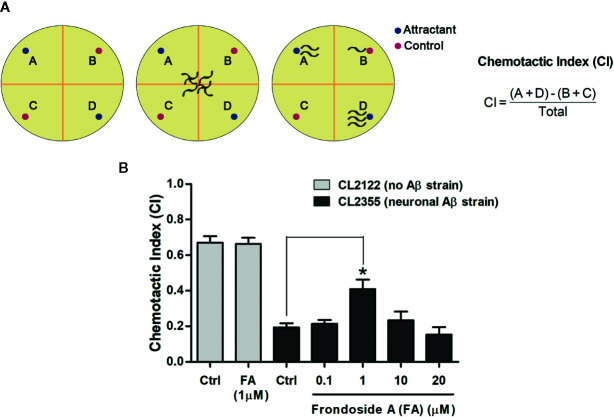
Assays for chemotaxis behavior in neuronal Aβ-expressing strain CL2355. **(A)** Schematic diagram of chemotaxis assay. After treatment, *C. elegans* CL2355 and CL2122 control strain were placed on the center of the assay plate and incubated for 1 h. The worms migrated to each quadrant (A&D with chemical attractant, B&C for control without attractant) were scored and calculated for chemotactic index (CI). **(B)** Data represent CI ± SD in neuronal Aβ strain CL2355 and CL2122 treated with vehicle or different concentrations of frondoside A ranging from 0.1 to 20 µM. The results are obtained from three independent experiments with 60 worms in each group (*p < 0.05 compared with control group).

In *C. elegans* strain CL2355, Aβ is expressed in pan-neuronal cells when induced by temperature up-shift, and this causes defect in chemotaxis response, which is mediated by a circuit of interconnected sensory and motor neurons (Hobert, 2003; Wu et al., 2006). Thus, we tested CL2355 and its control strain CL2122 with benzaldehyde, a chemical attractant, to evaluate their chemotaxis behaviors by determining CI values. CL2355 fed with only vehicle displayed a chemotaxis dysfunction as demonstrated by a signiﬁcant reduction of CI when compared with the control strain CL2122 (Ctrl CI_CL2122_, 0.67 ± 0.06 vs. Ctrl CI_CL2355_, 0.19 ± 0.03; three experiments; *p* < 0.001) (**Figure 6B**). Control CL2122 strain fed with frondoside A (at 1 µM) showed no change of their CI (0.67 ± 0.06 for CL2122 worms fed with vehicle and 0.66 ± 0.06 for CL2122 worms fed with 1 µM frondoside A). On the other hand, feeding with frondoside A could significantly normalize the reduced CI in the neuronal Aβ transgenic strain CL2355 (Ctrl CI_CL2355_, 0.19 ± 0.04 vs. frondoside A_1__µM_ CI_CL2355_, 0.41 ± 0.09; three experiments; *p* < 0.05) (**Figure 6B**). These findings suggested that frondoside A could alleviate Aβ neurotoxicity by attenuating its aggregation and consequently improving defective chemotaxis response.

## Discussion

A broad range of ginsenosides, saponins obtained from ginseng herbs, have been previously shown as effective compounds against Aβ toxicity in AD (Kim et al., 2013; Zhang et al., 2017). In the present study, we demonstrated for the first time the effects of frondoside A saponin from *C. frondosa* sea cucumber in attenuating Aβ toxicity in transgenic *C. elegans* model of AD by blocking its formation in the aggregation pathway. We found that *C. elegans* strain CL4176 treated with frondoside A at low concentrations (0.05-1 µM), starting from eggs, exhibited anti-paralytic effect in dose-dependent manner. The most significant delay of paralysis was observed in the worms treated with 1 µM. Moreover, this anti-paralytic effect of frondoside A was higher than ginsenoside-Rg3 at 50 µM (**Figures 1C, D**). As the same pattern of treatment, the anti-paralytic effect of frondoside A was slightly increased when tested concentration was raised up from 0.05 to 0.5 µM, and its concentration at 1 µM dramatically prevents late-onset paralysis that caused by constitutive expression of Aβ in CL2006 strain (**Figure 2**). These evidences supported that frondoside A at low doses was shown to potentially attenuate toxicity of Aβ expression in both inducible and constitutive manner. Moreover, the dose-response curve (**Figure 1E**) revealed that frondoside A especially at 1 µM protected the worms from Aβ-induced paralysis with the highest effect. Although the anti-paralytic effect of frondoside A was sequentially reduced at the higher concentrations (10 and 20 µM), no toxic effect of frondoside A in causing the death of the treated worms was observed even at a high dose of 20 µM. In cancer studies, high concentrations of frondoside A (at 100 or 800 µg/kg/day) were well tolerated with no signs of severe side effect on immune cells and red blood cells in mice (Al Marzouqi et al., 2011; Dyshlovoy et al., 2015). In addition, no changes in behavior, body weight, and weight of internal organs were detected in the treated mice. Consistent with these previous reports, we found that the worms remained unaffected by a relative high dose of frondoside A. Furthermore, in contrast to all treatment, administration of frondoside A before or after Aβ induction was not able to delay Aβ-induced paralysis (**Supplementary data**). These suggested that the metabolism of this compound in the worms was depend on its treatment time. Similar results were also obtained in the worms treated with EGb 761 (Wu et al., 2006), oleuropein aglycone (Diomede et al., 2013), and scorpion venom heat-resistant peptide (Zhang et al., 2016). Taken together, these findings implied there was an optimal time for frondoside A treatment for the drug to be effective in attenuating Aβ toxicity.

Based on *in vitro* studies it has been demonstrated that Aβ aggregation pathway starts from monomers, oligomers, protofibrils, and finally fibrils or plaques (Bitan et al., 2003; Urbanc et al., 2004). Aβ oligomers have been found to be the most toxic species in AD pathogenesis (Salminen et al., 2008; Larson and Lesné, 2012). Moreover, a number of studies implicated the presence of intracellular Aβ oligomers initiated early stage of AD (Kienlen-Campard et al., 2002; Umeda et al., 2015), which was correlated with early symptoms in AD patients (Kayed et al., 2003; Nimmrich and Ebert, 2009; Taylor et al., 2010). Consistent with previous observations, paralysis in *C. elegans* occurred before detectable Aβ deposition (Drake et al., 2003). Therefore, it is likely that the anti-paralytic effect of frondoside A in *C. elegans* would be through the suppression of pre-fibrillar Aβ formation. This is supported by our finding that the feeding of worms with frondoside A significantly reduced the level of toxic oligomeric species (Aβ band at 20 kDa) shown in **Figure 3**. Moreover, the levels of high molecular weight Aβ species at 25, 60, 75, 100, and 150 kDa were also significantly reduced in frondoside A-treated CL2006 worms, a constitutively expressing Aβ strain. Moreover, in this worm strain, a robust decrease of Aβ_3–42_-forming deposits that increases with age as appeared in humans (Diomede et al., 2013) (**Figure 4**) was also related with a significant decrease of high-order molecular weight Aβ species in the treated worms. However, frondoside A did not affect the amount of Aβ monomers, which are responsible for normal synaptic plasticity and memory in hippocampal formation (Garcia-Osta and Alberini, 2009; Puzzo and Arancio, 2013; Brothers et al., 2018). It is, therefore, suggested that suppression of paralysis by frondoside A is mediated primarily by the reduction of Aβ oligomers, the most toxic species. A study by Mu Zhang (Zhang et al., 2017) indicated that ginsenoside-Rg3 had the same effect as frondoside A in minimizing Aβ deposition, but the compound failed to prevent paralysis in worms. Probably, ginsenoside-Rg3 might inhibit the aggregation process at later stages than frondoside A. This implied that small Aβ oligomers was more toxic than their higher molecular weight aggregates and related to be the cause of paralysis, a pathogenic symptom of AD worms. Interestingly, previous studies have found that frondoside A saponin at subtoxic dose (0.2 µg per mouse) was able to strongly stimulate lysosomal activity in mouse macrophages and maintained this activity over 10 days *in vivo* (Aminin et al., 2008). This mechanism may help to explain the frondoside A action in preventing Aβ formation in worms, which should be further studied in the future.

Based on the amyloid cascade hypothesis, oxidative stress generated by Aβ may be one cause of AD pathogenesis (Butterfield, 1997). In transgenic *C. elegans*, ROS production is typically resulted from Aβ pre-fibrillar aggregates (Drake et al., 2003). Here, we found that frondoside A could suppress ROS production, suggesting that a significant decrease of ROS by frondoside A may be an indirect outcome from their suppressive effect on toxic oligomeric species of Aβ peptides. Interestingly, ginsenoside-Rg3, which had a lesser effect in delaying paralysis also displayed a lower decrease of ROS production in comparison with frondoside A that was significantly protect paralysis in the treated worms. These indicated that a strong effect in suppressing ROS is essential for prevention against paralysis, a defect symptom in Aβ-expressed worms. Since the known cause of worms with paralysis was oxidative stress-mediated by pro-fibrils of Aβ (Drake et al., 2003), it confirmed that the low effects in suppressing paralysis and ROS of ginsenoside-Rg3 treatment in worms may due to a later inhibition of the aggregation process when higher molecular weight fibrils are formed (Bitan et al., 2003; Urbanc et al., 2004). As well, frondoside A at a concentration of 1 µg/ml was found to inhibit generation of ROS in mouse peritoneal macrophages *in vitro* (Aminin et al., 2008). Based on this finding, a direct scavenging effect of frondoside A on superoxide production might also be another plausible ability of frondoside A. Nevertheless, both indirect and direct effects of frondoside A to suppress ROS production have benefits for AD treatment.

In addition to CL4176 and CL2006 strain whose muscle expressed Aβ, CL2355 strain, whose neurons expressed Aβ, was also tested for the effect of frondoside A on chemotaxis function. In this latter strain, chemotaxis dysfunction, a well-defined pathological behavior, was mediated by Aβ-induced toxicity (Wu et al., 2006). As reported in previous study, the scorpion venom heat-resistant peptide (SVHRP) that had inhibitory effect on small oligomeric forms of Aβ was shown to protect neuronal Aβ expression-induced defects in chemotaxis response (Zhang et al., 2016). Thus, we evaluated whether the suppressing effects of frondoside A on Aβ oligomers would protect neuronal system from Aβ-induced toxicity *in vivo*. Our result showed that frondoside A at a concentration of 1 µM significantly improved the chemotaxis behavior of CL2355 worms (**Figure 6**). This confirmed the ability of frondoside A against Aβ-induced neurotoxicity. Furthermore, administration of frondoside A at 0.2 µg/ml was found to downregulate HnRNP K, a RNA-binding protein, (Aminin et al., 2009), which consequently enhanced translation of p21 mRNA to induce neurite outgrowths from neurons in primary culture (Tanaka et al., 2002). These data suggested an additional action of frondoside A in promoting outgrowths of neuronal processes, which might be suppressed in the presence of intraneuronal Aβ oligomers (Umeda et al., 2015).

Chemically, both frondoside A and ginsenoside-Rg3 belong to triterpene glycoside or saponin group of compound whose structural composition comprises of a sugar moiety attached to a triterpene or steroid aglycone (Park et al., 2014). Similarly, these two compounds have saccharide chain linked to the C-3 position of the aglycone unit. However, types of aglycone and carbohydrate are quite different from each other. Frondoside A has an acetoxy group at C-16 of a lanostane derivative whereas aglycone of ginsenoside-Rg3 is tetracyclic dammarane skeleton. Ginsenoside-Rg3 has two glucopyranosyl sugar chains. In contrast, frondoside A is a pentaoside with xylose as the third monosaccharide residue and 3-O-methylglucose as the terminal monosaccharide residue and it contains a sulphate group on the ﬁrst sugar moiety (**Figure 1A**). As reported previously, monosulfated frondoside A at subtoxic doses was more active than their tetrasaccharide analogs in stimulating immunomodulatory effect of mouse macrophages (Aminin et al., 2010). It has been suggested that a sulfate group attached to penta-saccharide chain might be the crucial functional group of this sea cucumber saponin that enables it to be more effective in preventing Aβ oligomerization even at a low dose. Therefore, frondoside A may be a more efficient compound for treating AD than ginsenoside-Rg3. This notion needs further tests in higher animal model before frondoside A could be used for AD therapy in humans.

## Conclusion

In summary, we are the first to report on the suppressive effect of frondoside A against Aβ oligomerization and Aβ-induced toxicity at a low dose in transgenic *C. elegans*. Our data showed that frondoside A strongly protected the worms from Aβ-induced pathological behaviors including paralysis and chemotaxis dysfunction. Mechanistically, this compound reduced the level of various Aβ species that ranged from low to high molecular weights thus consequentially suppressed Aβ deposition, while it did not alter the expressing level of Aβ monomer, which has physiological functions. This Aβ aggregation suppressing effect was also correlated with ROS reduction in the worms. Additionally, frondoside A exhibited stronger effects than ginsenoside-Rg3, thus frondoside A could be used more effectively against Aβ toxicity and possibly in AD treatment. For the underlying mechanism of how frondoside A exerts its anti-Aβ aggregation, degradative pathway through autophagy-lysosome is our aim of interest to elucidate in the future.

## Data Availability Statement

The raw data supporting the conclusions of this article will be made available by the authors, without undue reservation, to any qualified researcher.

## Ethics Statement

The animal study was reviewed and approved by Mahidol University-Institute Animal Care and Use Committee.

## Author Contributions

TT designed, performed the all experiments, and analyzed the data. PS and KM interpreted the results and conceptualized the findings and contributed reagents/materials/analysis tools. TT and KM wrote the manuscript. PS edited the writing. All authors contributed to the article and approved the submitted version.

## Funding

This work was supported by the Royal Golden Jubilee (RGJ) Ph.D. scholarship to TT (PHD/0004/2558) and a research grant from Faculty of Science, Mahidol University to KM. *C. elegans* strains used in this work were provided by the CGC, which is funded by NIH Office of Research Infrastructure Programs (P40 OD010440).

## Conflict of Interest

The authors declare that the research was conducted in the absence of any commercial or financial relationships that could be construed as a potential conflict of interest.
